# Coordination of care in the Chinese health care systems: a gap analysis of service delivery from a provider perspective

**DOI:** 10.1186/s12913-016-1813-8

**Published:** 2016-10-12

**Authors:** Xin Wang, Stephen Birch, Weiming Zhu, Huifen Ma, Mark Embrett, Qingyue Meng

**Affiliations:** 1 School of Health Care Management, Shandong University, Jinan, China; 2 Department of Clinical Epidemiology & Biostatistics, McMaster University, Hamilton, Canada; 3 China Center for Health Development Studies, Peking University, 38 Xueyuan Road, Haidian district Beijing, China; 4 Health policy, Faculty of health science, McMaster University, Hamilton, Canada

**Keywords:** Organizational integration, System-level care coordination, System integration, Gap analysis

## Abstract

**Background:**

Increases in health care utilization and costs, resulting from the rising prevalence of chronic conditions related to the aging population, is exacerbated by a high level of fragmentation that characterizes health care systems in China. There have been several pilot studies in China, aimed at system-level care coordination and its impact on the full integration of health care system, but little is known about their practical effects. Huangzhong County is one of the pilot study sites that introduced organizational integration (a dimension of integrated care) among health care institutions as a means to improve system-level care coordination. The purposes of this study are to examine the effect of organizational integration on system-level care coordination and to identify factors influencing care coordination and hence full integration of county health care systems in rural China.

**Methods:**

We chose Huangzhong and Hualong counties in Qinghai province as study sites, with only Huangzhong having implemented organizational integration. A mixed methods approach was used based on (1) document analysis and expert consultation to develop Best Practice intervention packages; (2) doctor questionnaires, identifying care coordination from the perspective of service provision. We measured service provision with gap index, overlap index and over-provision index, by comparing observed performance with Best Practice; (3) semi-structured interviews with Chiefs of Medicine in each institution to identify barriers to system-level care coordination.

**Results:**

Twenty-nine institutions (11 at county-level, 6 at township-level and 12 at village-level) were selected producing surveys with a total of 19 schizophrenia doctors, 23 diabetes doctors and 29 Chiefs of Medicine. There were more care discontinuities for both diabetes and schizophrenia in Huangzhong than in Hualong. Overall, all three index scores (measuring service gaps, overlaps and over-provision) showed similar tendencies for the two conditions. The gap indices of schizophrenia (> 5.10) were bigger for diabetes (< 2.60) in both counties. The over-provision indices of schizophrenia (> 3.25) were bigger than diabetes (< 1.80) in both counties. Overlap indices for the two conditions exceeded justified overlaps, especially for diabetes. Gap index scores for schizophrenia interventions at the township-level and over-provision index scores for diabetes interventions at both village- and township-level showed big differences between the two counties. Insufficient medical staff with appropriate competencies, lack of motivation for care coordination and related supportive policies as well as unconnected information system were identified as barriers to system-level care coordination in both counties.

**Conclusion:**

Findings demonstrate that organizational integration in Huangzhong has not achieved a higher level of care coordination at this stage. System-level care coordination is most problematic at village-level institutions in Hualong, but at county-level institutions in Huangzhong. These findings suggest that attention be given to other aspects of integration (e.g., clinical and service integration) to promote system-level care coordination and contribute to the full integration of health care system in the pilot county.

**Electronic supplementary material:**

The online version of this article (doi:10.1186/s12913-016-1813-8) contains supplementary material, which is available to authorized users.

## Background

China’s health care delivery system is highly fragmented [1]. Since the introduction of the market economy, the role of government in financing and regulating the health care sector has been reduced [2]. In the rural county health care system, primary health care includes village clinics (VCs) and township health centers (THCs), while county hospitals (CHs) and institutions above the county level are intended to provide service on referral from village and township institutions. The majority of the hospitals need to earn revenues through the provision of health services, which encourages them to provide more profitable health care. Functions of the primary health care facilities include gate keeping, but this function does not operate well due to various reasons including a hospital-centered and fragmented health care delivery system [3]. Fragmentation includes the lack of coordination between different service levels and settings, duplication of services and infrastructure as well as health care provided inappropriately in relation to the institutions providing it [4]. Fragmentation often leads to access problems, poor services quality, inefficient use of resources, unnecessary increases in costs and low patient satisfaction [5]. Integrating and coordinating health care delivery at the system level is critical to containing cost escalation of medical care as well as improving the equity and efficiency of the health care systems.

Health care systems in China are facing considerable challenges of high disease burden and economic burden of chronic diseases, resulting from the rising prevalence of chronic conditions related to the aging population. The World Health Organization (WHO) reported that the impact on the Chinese economy of the accumulated loss of lives from heart disease, stroke and diabetes would be $556 billion during 2005–2015 in China [6]. Improvement to the effectiveness and efficiency of health care services through better system-level care coordination offers the potential to reduce this burden significantly. Individuals with chronic conditions, need flexible, continuous and seamless care, but instead often experience fragmented care from health professionals in different organizations, across different sites [7]. This problem of fragmentation is common to many health care systems in higher income countries that were designed primarily to deal with single, acute, and short-term illnesses [8].

WHO defines integrated care as “the management and delivery of quality and safe health services so that people receive a continuum of health promotion, disease prevention, diagnosis, treatment, disease-management, rehabilitation and palliative care services, through the different levels and sites of care within the health care system, and according to their needs throughout the life course [9].” The aim of integrated service delivery is providing the “right care” in the “right place” at the “right time”. A comprehensive approach to service delivery is needed to achieve integrated care and thus eliminate the fragmentation of care [10]. In the conceptual framework for integrated care, proposed by Valentijn et al. (2013), organizational integration is one of six dimensions to integrated care, which refers to the extent that services are produced and delivered in an inter-organizational way, to improve quality, market share and efficiency [11].

Leutz suggests “There are three levels of integration: linkage, coordination, and full integration.” [12] The current study focuses on the care coordination level, which is one step toward achieving full integration of the health care system. Haggerty et al. defined care coordination as the delivery of services by different care providers in a timely and complementary manner to achieve efficient and effect system integration [13]. This system-level care coordination (referred to simply as care coordination in the rest of the paper) is measured in terms of [14]: (1) the coordination process that is, communication across providers to provide coordinated care and (2) the coordination outcome, i.e., coordinated service delivery. Alter and Hage [15] argue that care coordination should be considered as a measure of system “performance”. In this paper, we measure care coordination from the perspective of service delivery. Many countries have attempted care coordination to achieve more efficient and effective integrated health care systems [14]. A review of studies on 26 Organization for Economic Co-operation and Development (OECD) countries concluded that coordination of care policies seemed likely to improve the quality of care [16]. Care coordination may lead to more appropriate care and improved efficiency, while under-coordination has been shown to increase risks, adverse events and costs [17]. The Integrated Health Service Delivery Network, developed by the Pan American Health Organization also emphasized the importance of coordinated care in health care systems [18].

Several pilot projects aimed to promote care coordination as a pathway to achieve the full integration of health care systems have been implemented in China in the last decade, especially after the current round of health system reform initiated in early 2009 [19]. Chinese government has tried to use the strategy of integrated health care to control the problems of chronic health conditions and cost escalation of medical care. To strengthen organizational integration of health providers and role of primary health providers in gatekeeping functions are the majority policy intervention. Existing research focuses on theoretical explorations and the introduction of experiences in other countries or areas including qualitative studies [1] that describe the design and process of integration among hospitals at different levels [20]. Others include evaluations with only limited empirical indicators, such as bed occupancy rates, revenues, and joint training programs among others [21]. However, little is known about providers’ perspectives regarding the impacts pilot studies have had on care coordination. Such knowledge is essential if care coordination is to contribute to the full integration of health care system.

**Fig. 1:**
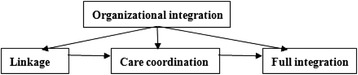
Interpretation of conceptual model The conceptual model is based on Leutz’s three levels of integration: linkage, coordination, and full integration. This study focuses on the care coordination level, which is one step toward achieving full integration of the health care system

The aims of the present study are to: (1) assess the effects of organizational integration on care coordination for patients with one of two tracer conditions; and (2) identify the barriers to care coordination and hence full integration of county health care systems in rural China. Figure 1 presents the conceptual model of this study.

## Methods

### Study settings

Qinghai Province implemented integration policies at the provincial level in 2013. Doctors in VCs are the first point of contact and serve as a gatekeeper to other institutions. VC doctors refer patients to CHs or THCs for appropriate services. The provincial ministry of health provides considerable resources to develop information technology to support and promote such referral systems [22].

Huangzhong County established three consortia for their medical institutions based on their levels and locations. Each consortium consists of one CH, several THCs and many VCs. Each CH is responsible for the management of general affairs in its consortium, including organizing routine meetings, sharing health care resources, conducting joint training, collecting information, and receiving patients referred from other institutions, among others. THCs and VCs are expected to comply with the management of the CH and refer patients within the consortium. A leadership group at the county level is responsible for coordinating the whole county health care system. As the county that implemented organizational integration, Huangzhong provided the focus for this study. To assess the effect of organizational integration on care coordination, we chose Hualong (also located in Qinghai Province) as a comparator county for two reasons. First, no organizational integration occurred in Hualong, and no referral policy was implemented. It remained a traditional rural three-tier health care system, with no mandatory restrictions on the point of entry and patient referral. Second, Hualong had levels of health resources and adoption of other health reform policies similar to Huangzhong.

### The tracers

Schizophrenia and diabetes were selected as tracer conditions for three reasons. First, care coordination in primary health care is of most value to patients with complex chronic conditions like schizophrenia and diabetes as they require a range of interventions that may change over time as the condition progresses [23]. Second, schizophrenia and diabetes have high prevalence and disease burdens and hence offer potential for substantial changes in disease burden. Third, the Chinese Health and Family Planning Commission project on equalizing public health services for all includes regulations relating to diabetes and schizophrenia [24].

### Study design and sampling design

This study used a mixed methods approach, combining quantitative and qualitative data collection and analyses organized in three phases: (1) a qualitative phase, aimed at developing Best Practice care pathways (service/institutional setting combinations) for diabetes and schizophrenia patients; (2) a quantitative survey, aimed at measuring reported actual practice for the two patient groups for comparison with the Best Practice for two conditions; and (3) semi-structured interviews with Chiefs of Medicine, to explore barriers to care coordination.

We chose mixed sampling methods to select institutions within the county health care systems. All institutions at the county level, including the health administration department, the center for disease control and prevention, the county hospital, the Chinese medicine hospital as well as maternity and child care centers, were included in the analysis. However, because of the much larger numbers of THCs and VCs, stratified samples of 3 THCs and 6 VCs in each county were included based on their geographical distance to the nearest upper-level institution.

### Phase 1: Development of the best practice

We developed disease-specific Best Practice intervention/level packages for schizophrenia and diabetes before conducting the service delivery survey, in order to provide questionnaires for the survey and compare survey results with best practice.

We developed a preliminary checklist for each disease based on national clinical pathways, prevention guidelines and other regulations related to schizophrenia [25] and diabetes [26]. Based on the stages of disease [16], we grouped interventions into prevention, screening, diagnosis, treatment, rehabilitation and case management. Case management was defined to include information management, follow-up, assessment of condition and consultation for post-treatment follow up in accordance with the health care system context for China.

We conducted consultations with experts to develop the Best Practice based on the preliminary checklist. We selected twenty senior doctors all members of the Chinese Society of Psychiatry or the Chinese Diabetes Society, for expert consultation by email. The consultation form contained the preliminary checklist of interventions and left space for doctors to add further interventions. Furthermore, we asked the doctors to match the interventions with the appropriate institution level. (See Additional file 1: Table S1 and Table S2)

### Phase 2: Analysis of care coordination

#### Data collection

We collected data for care coordination with questionnaires provided to key informant doctors in the relevant departments (Internal Medicine, Chronic Disease or Psychiatry) in each institution about current practice for patients with diabetes or schizophrenia. Chiefs of Medicine chose the key informant doctors. The questionnaire consists of a checklist of interventions previously identified as Best Practice and opportunities were provided for respondents to add further interventions. Trained investigators guided all questionnaires and interviews.

#### Data analysis

We used gap analysis to understand the discrepancy between current and best practice [27] using three indices: the gap index, the overlap index and the over-provision index.

Gap refers to instances where interventions would be provided by institutions at certain levels under Best Practice, but are not provided at those levels in actual practice. The gap index measures the mean number of inaccessible intervention “gaps” among all institutions, measured at either a specific institutional level or for the whole county health care system. So with intervention gaps at the township level, patients have to seek health services that ought to be provided at the township level but are only provided from higher level institutions, which results in higher health care expenditures to the system and higher costs to the patient [3].

Overlap refers to the same interventions being provided by institutions at multiple levels. Given the diverse entry points for interventions and specific nature of medical professions, some overlaps may be warranted (i.e., justified overlap [28]) while other overlaps are unjustified. The difference between total overlap and justified overlap increases with inappropriate duplication of existing services in the county health care system, and hence is a measure of inefficiency in service delivery [29].

The overlap index measures the proportion of all interventions that are provided by institutions at two or more levels. A, B and C are the sets of care actually provided by institutions from village-level, township-level, and county-level respectively. Based on the algorithms of sets, union (∪) is for all non-duplicated elements in different sets and intersection (∩) is for the same elements in different sets. For example, A ∩ B represents the number of types of interventions provided by both village-level and township-level institutions. A ∪ B represents the number of types of interventions provided by either VC or THC or both of them. The overlap index between VCs and THCs was calculated by the ratio of the number of interventions provided by both VCs and THCs to the number of all interventions provided by either VCs or THCs or both. The calculation of the justified overlap index is the same as above, except replacing “provided” by “expected to be provided under the Best Practice”.

$$ \mathrm{System}\;\mathrm{overlap}\;\mathrm{index}=\left(A\cap B\right)\cup \left(B\cap C\right)\cup \left(A\cap C\right)/\left(A\cup B\cup C\right) $$

$$ \mathrm{Overlap}\;\mathrm{index}\;\mathrm{between}\;\mathrm{V}\mathrm{C}\mathrm{s}\kern0.24em \mathrm{and}\;\mathrm{THCs}=\left(A\cap B\right)/\left(A\cup B\right) $$

$$ \mathrm{Overlap}\;\mathrm{index}\;\mathrm{between}\;\mathrm{THCs}\;\mathrm{and}\;\mathrm{C}\mathrm{H}\mathrm{s}=\left(B\cap C\right)/\left(B\cup C\right) $$

Over-provision refers to instances where Best Practice requires that interventions not be provided by institutions at specific levels, but such interventions under current practice are provided at those levels. The over-provision index measures the mean number of interventions actually provided by institutions at a certain level or the whole health care system, which would not be provided by these institutions under a Best Practice model. The over-provision of interventions, often driven by profit and competition among institutions, has been shown to lead to poor quality health services [30].

The hypothesis of this study is that organizational integration in Huangzhong will have achieved higher levels of care coordination than observed in Hualong, a county with no organizational integration policy. This higher level of care coordination would be indicated by smaller index scores for gap and over-provision, and a smaller difference between the index score for overlap and the index score for justified overlap.

### Phase 3: Factor analysis of care coordination

#### Data collection

Semi-structured interviews were conducted with Chiefs of Medicine in each surveyed institution to collect data about barriers to care coordination in the local health care system. The interview (on average 35 min) focused on three questions: 1) what is the current level of care coordination among providers of different level in the county health care system? 2) What are the barriers to achieving increased care coordination in the county health care system?; and 3) How might those barriers be eliminated? All interviews were conducted in Chinese, the first language of both interviewers and interviewees.

#### Data analysis

Interviews were transcribed, and thematic analysis was conducted using MAXQDA 11 to identify barriers to care coordination.

### Other methodological issues

The survey was conducted in August and September, 2014, nine months after the implementation of organizational integration in Huangzhong. To ensure a high response rate, the staff of county health bureau accompanied the researchers to the institutions to make introduction to the chiefs of medicine and provide official support for the research study. County Health Bureau staffs were not present during the interviews and were not provided with details of any individual’s responses.

## Results

### Sample size

Twenty nine institutions from two counties were surveyed. The types and numbers of institutions in each county are shown in Table 1.

**Table 1:** No. of institutions in two counties

Levels	Types of institutions	Huangzhong		Hualong	
		Surveyed	In total^a^	Surveyed	In total^a^
County level	Health Administration Department	1	1	1	1
	Center for disease control and prevention	1	1	1	1
	County hospital	2	2	1	1
	Chinese medicine hospital	1	1	1	1
	Maternity and child care center	1	1	1	1
Township level	Township health center	3	14	3	19
Village level	Village clinics	6	395	6	362

^a^The total number of each type of institution in the county

Among experts selected from the Chinese Society of Psychiatry and the Chinese Diabetes Society for expert consultation of Best Practice, thirteen doctors (65 %) for schizophrenia and 11 doctors for diabetes (55 %) responded to the questionnaire. These responses were used to arrive at the best practice models of what to provide and at what level for the two conditions.

The sample size for doctors is shown in Table 2. The response rate was 100 %. As the numbers of institutions varies in each county and not all institutions are able to provide interventions for both conditions, the sample sizes are not the same in both counties.

**Table 2:** Sample size of doctors and Chiefs of Medicine

	No. of doctor (schizophrenia)	No. of doctor (diabetes)	No. of Chiefs of Medicine
Huangzhong	10	12	15
Hualong	9	11	14

Twenty nine Chiefs of Medicine, one from each institution, were interviewed.

### Service delivery coordination from the health professionals’ perspective

Using McDonald’s framework [31], care coordination was measured using three perspectives: the system, the health care professional, and the patient/family. In this study, a health care professional perspective was adopted and care coordination was measured by the continuity of interventions in the whole health care system and coordination of different types’ interventions at different institution levels.

#### Discontinuity of interventions in the whole health care systems

**Fig. 2:**
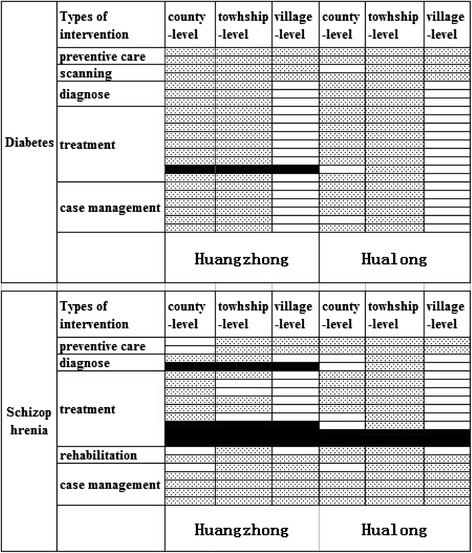
Maps of interventions provided in both counties. The top half of the figure shows the interventions for diabetes provided by the three levels of institutions in both counties, and the bottom half shows the interventions for schizophrenia. Each row represents an intervention in standard health service package for two conditions (see Additional file 1: Table S1 and Table S2). Each column represents one institutional level. Blank cell represents provided intervention. Cell with spots represents unavailable intervention. Cell in black represents intervention discontinuity of the health care system

In earlier studies, seeking health services from higher level hospitals outside of the county was found to result in higher patient out of pocket costs because medical insurance provides lower reimbursement rates for out of county services [32]. It is necessary therefore to provide a full range of interventions within each county health care system to avoid discontinuity of care in the county health care system. Figure 2 (Title: Maps of interventions provided in both counties) shows the interventions for two conditions provided by the three levels of institutions in both counties. The absence of surgical treatment for type II obesity diabetes in Huangzhong resulted in a discontinuity in care.. For schizophrenia patients, neither county provides psychological treatments or “other physical therapies”. Also, physiotherapy and psychological assessment are not provided in Huangzhong.

#### Coordination of different types of interventions at different institutional-levels

**Fig. 3:**
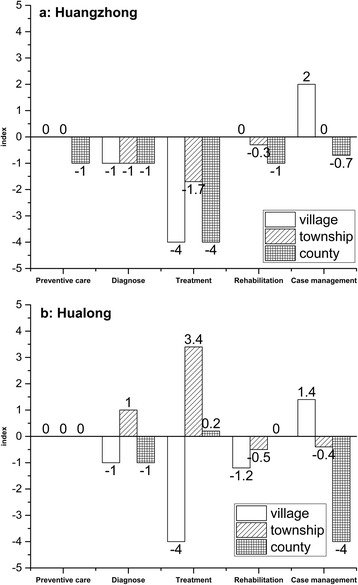
Pannel a shows gaps and over-provisions of six types interventions related to schizophrenia provided by different levels in Huangzhong, while pannel b shows that in Hualong. The horizontal axis represents the type of interventions. The vertial axis represents gap index (below 0.0) and over-provision index (above 0.0). Bars in different formats represent different institutional levels

**Fig. 4:**
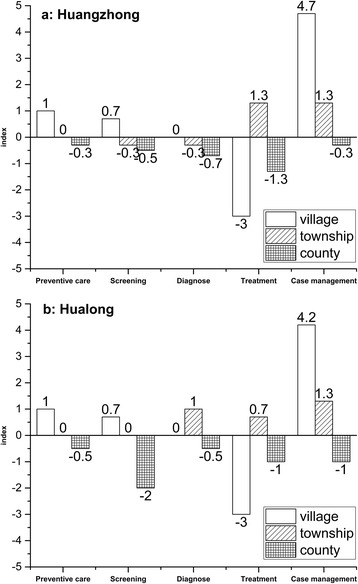
Pannel a shows gaps and over-provisions of six types interventions related to diabetes provided by different levels in Huangzhong, while pannel b shows that in Hualong. The horizontal axis represents the type of interventions. The vertial axis represents gap index (below 0.0) and over-provision index (above 0.0). Bars in different formats represent different institutional levels

Table 3 presents index scores for gap, overlap and over-provision for the two conditions in the three levels of institutions as well as at the system level in both counties. Differences of index scores between the two conditions are similar in both counties. For example the gap index score for schizophrenia is larger than for diabetes in both counties. At the system level, overlap index scores for the two conditions exceed justified overlap, especially for diabetes. The gap and over-provision of each type of intervention for the two conditions are presented in Figs. 3 and 4.

**Table 3:**
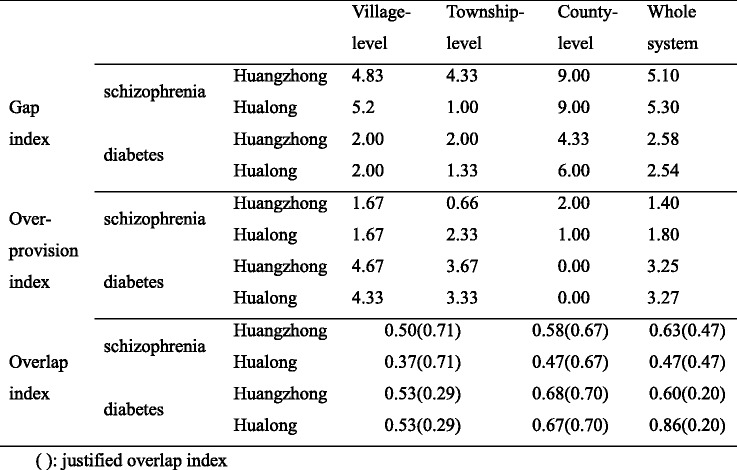
Gap index, overlap index and over-provision index of two chronic diseases in both counties

Figure 3a shows service delivery of each type intervention for schizophrenia in Huangzhong, compared to Best Practice. Only the CH has a gap for preventive care. Gaps for diagnostic interventions and treatment are common to all levels of institutions. Although there is a gap for case management at the county level, this contrasts with over-provision of case management at the village level.

As illustrated in Fig. 3b, Hualong institutions at all three levels delivered appropriate preventive interventions. There are gaps for diagnostic interventions at both VCs and CHs, but diagnostic over-provision at THCs. VCs did not provide any treatment, but both THCs and CH had over-provision of treatment. Gaps for rehabilitative interventions are observed at both village and township levels respectively, as well as gaps in case management at the CH level.

In Fig. 4a, over-provision is found at the VHC but a gap at the CH level for preventive interventions. According to the Best Practice, not all institutions at township and county levels should provide screening and diagnostic interventions. Regarding treatment, both VCs and CHs have gaps. However, case management showed large over-provision index scores, rather than gaps.

The delivery models for the five types of interventions for diabetes in both counties are quite similar (see Fig. 4). In comparison with Hauangzhong (Fig. 4a), THCs in Hualong (Fig. 4b) do not have gaps for screening and diagnostic interventions. At the same time, CHs in Hualong did not do as well as Huangzhong for screening and case management, as indicated by the larger gap index scores in Hualong.

### Barriers to care coordination in both county health care systems

Interviews with Chiefs of Medicine indicate some common barriers to care coordination in both counties, as well as some barriers specific to each county. The following are participants’ perceived barriers to care coordination with supporting quotations (translated from Chinese) from interviews.

The first common barrier is insufficient medical staff with appropriate competencies to perform the respective duties, as well as high rates of staff turnover. “*According to the provincial regulation (1.12 medical staff per 1,000 people), 386 medical staff are needed in our county, but we have less than 286 medical staff”.—Leader of the Health Department in Hualong*. For example, village doctors are expected to provide 13 categories of preventive care, but many village doctors in Hualong are not qualified to provide these services. As a result, THCs provide these services even though it would be more appropriate for VCs to provide them, which, in turn, adversely impacts the THC’s capacity to meet its own responsibilities.

The second common barrier is the lack of motivation for care coordination. Most Chiefs of Medicine reported that their priorities were to promote the development of their own institutions to compete with other institutions, which limited their interest in pursuing care coordination with each other, especially among CHs. The reasons for this were twofold. First, coordination often requires the CH to provide technical support to the other institutions but CHs do not have sufficient staff to satisfy this requirement. Second, there is no incentive mechanism to support such coordination. The absence of policies on performance evaluation and rewards in Hualong did not encourage or reward care coordination. There were policies on performance evaluation and rewards in consortia in Huangzhong, though these were not well implemented. The officials interviewed in Huangzhong suggested that there was more cooperation among institutions in the same consortium than before, but there still was much room for improvement.

The third common barrier is the lack of supporting policies. The medical insurance scheme provides reimbursement rates that differ only marginally between institution levels and hence do not adequately guide patients to the appropriate level for services. The last common barrier is the unconnected health information system, especially the poor linkages between the county-level and the other two levels, which hampers continuity of patient information and cross-level care coordination.

In addition to the common barriers, each county system had other barrier. The absence of THCs in the downtown area of Huangzhong has left the county hospitals to undertake 13 categories of preventive care and case management interventions. This may limit CHs’ capacity to deliver other interventions while capacity is being used to provide these health centre responsibilities. In Hualong, policy makers need to pay more attention to meeting the primary care expectations of each institution. VCs are most in need of improvement. “*Most village doctors were transformed from health workers before the foundation of P.R. of China (1949), now they are old and many can’t prescribe or deal with emergency situations.*”*—Leader of the Center for disease control and prevention in Hualong*.

## Discussion

The study illustrates that organizational integration alone is not associated with a higher level of system-level care coordination in the case of schizophrenia and diabetes patients in one Chinese county. Gap analysis was useful to present policy makers a comprehensive overview of care coordination from the perspective of providers, with gaps, over-provision and overlap of health care. Interviews with Chiefs of Medicine shed light on the barriers to care coordination.

Results of the quantitative analysis did not show a significantly higher degree of care coordination in Huangzhong (with organizational integration) than in Hualong (without organizational integration). However, the results displayed different shortcomings in the two County health care systems. The greatest weakness related to the VCs in Hualong but to CHs in Huangzhong. The interventions provided by THCs in Hualong were closer to Best Practice than in Huangzhong, with a lower gap index score. According to the interviews, in Hualong, where organizational integration had not occurred, there were policies on support from CHs and provincial hospitals to THCs. Accordingly, the implementation of these policies enhanced the service delivery ability of THCs. Overall, there were lower gap index scores and higher over-provision index scores in Hualong than in Huangzhong for both conditions. Regarding overlap, although the index score in Huangzhong is closer to Best Practice than in Hualong, there is much room for improvement in both counties. All three index scores showed similar tendencies for the two conditions.

There was no clear evidence from the interviews indicating a higher level of care coordination in Huangzhong than Hualong. But the officials interviewed in Huangzhong suggested there were positive impacts on cooperation among institutions in each medical consortium. For instance, it is easier for patient referral and expert consultation, which is consistent with the result of some previous studies [33]. Our results are supported by some studies [34], even though they are not consistent with findings from other studies that care coordination can be achieved after organizational integration [14]. These results may result from: (1) organizational integration’s impacts on service delivery being delayed beyond the period of follow up in our study; (2) Organizational integration being a necessary but no sufficient condition for changing provider behavior.

There are several limitations to this exploratory study. Firstly, the methodology does not allow us to demonstrate causality between organizational integration and care coordination. Deeper qualitative analysis is needed to provide evidence for the nature of their relationship. Gap analysis in this study analyzed complementarity among different providers on service delivery, which was one aspect of care coordination. But it could not measure the dynamic process of care coordination. The development of best practices is one step for gap analysis, making preparation for comparing with the current situation, instead of guiding doctors to provide services for patients. It is inappropriate to apply them to some patients, who experience multiple co-morbidities besides diabetes or schizophrenia. The second limitation concerns the different number of institutions in the two counties and the small number of some types of interventions for the two conditions. For example, there is only one county-level hospital in Hualong. There are more treatment and case management interventions than preventive, diagnostic and rehabilitative interventions. The index we calculated was based on absolute numbers. Therefore, the smaller gap index scores for preventive or diagnostic interventions does not mean a smaller problem than for treatment and case management interventions. Further research might benefit from more interventions of each type and calculation based on the percentage of interventions. The third limitation is Huangzhong may have been observed before the full effects of organizational integration occurred after nine months of implementation. Further study is required to explore dynamic changes in care coordination. The fourth limitation is that the study has focused exclusively on the perspective of providers. In addition, the data about service delivery was self-reported, which might introduce a significant bias. Facing the fragmentation of service delivery in rural Chinese health care systems, patients’ perspectives on level and determinants of and barriers to care coordination would provide important additional insights for decision-makers. Finally, the study was concerned with the types of interventions provided, without taking the quality of care into account.

## Conclusions

This study makes two contributions. First, gap analysis provides a methodology for broad use in health care systems to provide evidence on system-level care coordination from the perspective of providers. It can be used in other health care systems in China and other low-and-middle income countries, experiencing organizational integration. Second, gap analysis identified barriers to bridging gaps, reducing over-provision and avoiding overlap of interventions in both counties.

Care coordination is dependent on local health care system settings with developments towards care coordination varying in each health care system. Although there is evidence that organizational integration has a positive impact on integrated care, it may not be an essential step in other health care systems. Organizational integration has been unable to achieve care coordination in Huangzhong. It maybe that care coordination requires other aspects of integration (e.g., clinical and service integration in Valentijn’s conceptual framework [11, 35]) at the point of delivery.

## Additional file

Additional file 1: Standard health service packages for schizophrenia and diabetes in a county health care system. (DOC 69 kb)

## Abbreviations

CH: County hospital
OECD: Organization for Economic Co-operation and Development
THC: Township health center
VC: Village clinics
WHO: World Health Organization
