# Supplementary material for: Coordination of care in the Chinese health care systems: a gap analysis of service delivery from a provider perspective
Source: BMC Health Serv Res. 2016 Oct 12;16:571. doi: 10.1186/s12913-016-1813-8 (PMC5062922; doi:10.1186/s12913-016-1813-8)
Supplement: Additional file 1: — Standard health service packages for schizophrenia and diabetes in a county health care system. (DOC 69 kb) [file 12913_2016_1813_MOESM1_ESM.doc]

**Additional file 1**

**Table S1 Standard health service package for schizophrenia in a county health care** system

|  | Services | County  -level | Township  -level | Village  -level |
| --- | --- | --- | --- | --- |
| **1** | **Prevention** |  |  |  |
| 1.1 | Education on psychological and mental health | √* | √ | √ |
| **2** | **Diagnosis** |  |  |  |
| 2.1 | Taking medical history | √ | √ | √ |
| 2.2 | Physical examinations |  | √ | √ |
| 2.3 | Psychiatric examination |  |  | √ |
| 2.4 | Psychological assessment |  |  | √ |
| **3** | **Treatment** |  |  |  |
| 3.1 | Outpatient treatment | √ | √ | √ |
| 3.2 | Acute exacerbation treatment |  |  | √ |
| 3.3 | Treatment for chronic patients | √ | √ | √ |
| 3.4 | Drug treatment | √ | √ | √ |
| 3.5 | Anti-psychotic drug treatment monitoring |  |  | √ |
| 3.6 | Operative treatment |  |  |  |
| 3.7 | Physiotherapy |  |  | √ |
| 3.8 | Psychological treatments | √ | √ | √ |
| 3.9 | Other physical therapies |  |  | √ |
| **4** | **Rehabilitation** |  |  |  |
| 4.1 | Hospital rehabilitation | √ | √ | √ |
| 4.2 | Community rehabilitation | √ | √ | √ |
| **5** | **Case management** |  |  |  |
| 5.1 | Patient-related information management | √ | √ | √ |
| 5.2 | Follow up and risk assessment (at lease 4 times/year) |  | √ | √ |
| 5.3 | Intervention based on follow-up |  | √ | √ |
| 5.4 | Provide rehabilitation guideline for patients | √ | √ |  |
| 5.5 | Provide psychological support for relatives | √ | √ | √ |

*: All check marks in the table means that institutions at the certain level should provide the specific service.

**Table S2 Standard health service package for diabetes in a county health care** system

|  | Services | County  -level | Township  -level | Village  -level |
| --- | --- | --- | --- | --- |
| **1** | **Prevention** |  |  |  |
| 1.1 | Education of preventive knowledge | √* | √ | √ |
| 1.2 | Consultation for high-risk population |  | √ | √ |
| **2** | **Screening** |  |  |  |
| 2.1 | Screening for general population | √ | √ | √ |
| 2.2 | Screening for high-risk population |  | √ | √ |
| **3** | **Diagnose** |  |  |  |
| 3.1 | Fasting plasma glucose detection (FPG) |  | √ | √ |
| 3.2 | Oral glucose tolerance test (OGTT) |  |  | √ |
| 3.3 | A1C |  |  | √ |
| **4** | **Treatment** |  |  |  |
| 4.1 | Medical nutrition therapy |  | √ | √ |
| 4.2 | Exercise therapy | √ |  | √ |
| 4.3 | Drug treatment |  | √ | √ |
| 4.4 | Comprehensive treatment |  | √ | √ |
| 4.5 | Treatment of low blood glucose | √ | √ | √ |
| 4.6 | Treatment of chronic complications |  |  | √ |
| 4.7 | Treatment of acute complications |  | √ | √ |
| 4.8 | Surgical therapy |  |  |  |
| 4.9 | Treatment of special cases |  |  | √ |
| **5** | **Case management** |  |  |  |
| 5.1 | Patient-related information management | √ | √ | √ |
| 5.2 | Follow up (at lease 4 times/year) |  | √ | √ |
| 5.3 | Assessment and adjust of treatment |  | √ | √ |
| 5.4 | Physical examination (at least once a year) |  |  | √ |
| 5.5 | Education on patient self-management |  | √ | √ |

*: All check marks in the table means that institutions at the certain level should provide the specific service.
